# Exploring Individual Differences in Online Addictions: the Role of Identity and Attachment

**DOI:** 10.1007/s11469-017-9768-5

**Published:** 2017-05-03

**Authors:** Lucia Monacis, Valeria de Palo, Mark D. Griffiths, Maria Sinatra

**Affiliations:** 1 Universita degli Studi di Foggia, Foggia, Italy; 2 International Gaming Research Unit, Division of Psychology, Nottingham Trent University, Nottingham, NG1 4BU UK; 3 Department of Educational Sciences, Psychology, Communication, University of Bari Aldo Moro, Bari, Italy

**Keywords:** Behavioral addictions, Online addictions, Technological addictions, Identity styles, Attachment styles

## Abstract

Research examining the development of online addictions has grown greatly over the last decade with many studies suggesting both risk factors and protective factors. In an attempt to integrate the theories of attachment and identity formation, the present study investigated the extent to which identity styles and attachment orientations account for three types of online addiction (i.e., internet addiction, online gaming addiction, and social media addiction). The sample comprised 712 Italian students (381 males and 331 females) recruited from schools and universities who completed an offline self-report questionnaire. The findings showed that addictions to the internet, online gaming, and social media were interrelated and were predicted by common underlying risk and protective factors. Among identity styles, ‘informational’ and ‘diffuse-avoidant’ styles were risk factors, whereas ‘normative’ style was a protective factor. Among attachment dimensions, the ‘secure’ attachment orientation negatively predicted the three online addictions, and a different pattern of causal relationships were observed between the styles underlying ‘anxious’ and ‘avoidant’ attachment orientations. Hierarchical multiple regressions demonstrated that identity styles explained between 21.2 and 30% of the variance in online addictions, whereas attachment styles incrementally explained between 9.2 and 14% of the variance in the scores on the three addiction scales. These findings highlight the important role played by identity formation in the development of online addictions.

Over the last two decades, the increase in individuals engaging in online activities has led psychologists and researchers to study the more problematic side of excessive technology use (i.e., online addictions; Kuss et al. 2014). Scientific interest in this topic began in the mid-1990s with papers by Griffiths (1995, 1996, 1998) and Young (1996, 1998) who both developed criteria (primarily based on those for pathological gambling) to distinguish normal from addictive Internet usage. Young and Griffiths both noted symptoms such as increased investment of time spent on online activities, unpleasant feelings (anxiety, depression, emptiness) when offline, an increasing tolerance to the effects of being online, and the denial of the problematic behaviors. The similar symptomatology between problematic use of Internet, pathological gambling, and substance use disorders has been empirically supported (Brezing et al. 2010; Goldstein and Volkow 2011; Grant et al. 2010; Griffiths 2011; Kormas et al. 2011; Montag et al. 2012).

Two decades on, Internet addiction is considered by some (e.g., Kuss and Billieux 2016) to be an umbrella term that comprises a wide range of problematic online activities for individuals (e.g., online gaming, online gambling, online sex, social networking, etc.). Young (1999) classified Internet addiction into five sub-types (i.e., computer addiction, information overload, net compulsions, cybersexual addiction, and online relationship addiction) although this was heavily criticized by Griffiths (1999, 2000) who argued that most of these sub-types described addictions *on* the internet rather than *to* the internet (i.e., the internet was simply a medium that fueled other addictions such as gambling and gaming). Of these online problematic behaviors, Internet gaming disorder (IGD) has recently been included in the Section III (“Emerging measures and models”) of the latest (fifth) edition of the *Diagnostic and Statistical Manual of Mental Disorders* (DSM-5; American Psychiatric Association [APA] 2013). Perhaps more importantly, IGD was classified as a potential behavioral addiction following the reclassification of gambling disorder from a disorder of impulse control to a behavioral addiction.

Since the publication of the DSM-5, much research has been carried out into IGD. This has included the criteria proposed for the clinical diagnosis of IGD and has led to much debate in the literature (e.g., Petry et al. 2014; Griffiths et al. 2016). Underpinned by Griffiths’ (2005) biopsychological addiction components model, new instruments have been developed using the DSM-5 criteria such as the IGD-20 (Pontes et al. 2014) and the IGD9-Short Form (Pontes and Griffiths 2015) which have been validated in a number of languages including Italian, Spanish, Portuguese, and Persian (Fuster et al. 2016; Monacis et al. 2016a; Pontes and Griffiths 2016; Wu et al. 2017).

Griffiths’ (2005) addiction components model comprises six criteria: salience, mood modification, tolerance, withdrawal symptoms, conflict, and relapse. *Salience* occurs when addictive activities dominate a person’s thinking, feelings, and behavior; *mood modification* occurs when a person uses substances or is engaged in activities to change their mood state; *tolerance* refers to the need to increase (over time) the amounts of engagement in the addictive behavior to achieve the initial mood-modifying effects; *withdrawal symptoms* refer to the unpleasant feeling states and/or physical effects that occur when the individual decreases or suddenly reduces their addictive activities; *conflict* refers to both the intrapsychic and interpersonal problems that arise as a consequence of addictive activities and conflicts with all other things in their lives such as relationships and work and/or education; and *relapse* refers to the unsuccessful efforts to stop engaging in the addictive behavior if the individual is trying to cease.

The problematic use of social media (i.e., social networking site [SNS] addiction [Kuss and Griffiths 2017]) is another emerging technological addiction that has been argued as falling into Young’s (1999) online relationship addiction category (Kuss and Griffiths 2011). However, if social networking is seen as a discrete online application, it could arguably classed under her category of ‘net compulsions’ along with activities such as online gambling or gaming. Griffiths’ (2005) six criteria of addiction have also applied to SNS addiction, which have been operationalized by some psychometrically robust scales including the six-item Bergen Facebook Addiction Scale (BFAS; Andreassen et al. 2012) and the Facebook Intrusion Questionnaire (FIQ; Elphinston and Noller 2011). However, Griffiths (2012, 2013) argued that these instruments focused on just one specific commercial SNS (i.e., *Facebook*) rather than on the activity itself (i.e., social networking) and proposed that researchers should develop more reliable and valid addiction scales assessing social networking. This led to the development of the Bergen Social Media Addiction Scale (BSMAS; Andreassen et al. 2016).

To gain a better understanding of the online addictions, ongoing research has identified the risk factors related to the development of pathological behaviors, as well as the protective factors that appear to prevent such pathological behaviors. Empirical studies have highlighted an interplay of factors from intraindividual components to sociodemographic and personality-related characteristics (Andreassen 2015; Andreassen et al. 2013; Cerniglia et al. 2016; Kuss and Griffiths 2011, 2012; Wittek et al. 2016). Among dispositional determinants, identity characteristics have been found to be both protective factors and risk factors in health-risk outcomes, such as delinquency and substance abuse (Côté and Levine 2002). Marcia’s (1966) ego-identity status model states that individuals can use drugs for curiosity, as avoidant coping strategies and/or as an expression of an antisocial identity (Christopherson et al. 1988). Nevertheless, there has still been little attention regarding the relationship between identity and online addictions.

Given that Berzonsky’s social cognitive perspective of identity has already been applied to research on substance addictions (Hojjat et al. 2015; White et al. 1998; White et al. 2003), it may also provide a good framework to explore online addictions. More specifically, Berzonsky (1989, 1990) conceptualized identity as three styles referring to the strategy an individual uses to process, structure, utilize, and revise self-relevant information. *Information-oriented* individuals, characterized by a well-differentiated and well-integrated identity structure, actively seek out and deliberately process identity-relevant information to make well-informed choices. *Normative-oriented* individuals, characterized by conservative and inflexible attitudes, focus on the expectations, values, and prescriptions held by significant others, especially parents. *Diffuse-avoidance oriented* individuals, characterized by a fragmented and loosely integrated identity structure, procrastinate identity conflicts and problems until situational demands force them to make a choice. As these stylistic strategies process information from the social reality in which individuals reside, they may—to some extent—influence the way in which individuals deal with and behave in interpersonal relationships (Berzonsky 2011).

To the best of the present authors’ knowledge, very few studies have focused on identity styles as predictors of internet and social networking addiction. Normative and diffuse-avoidant styles have resulted in protective and risk factors respectively, whereas the informational style has resulted as an ambiguous factor, being both negatively associated to internet addiction (Arabzadeh et al. 2012; Ceyhan 2010; Tabaraei et al. 2014) and unrelated to internet addiction (Morsünbül 2014; Sinatra et al. 2016).

Another important but understudied factor in predicting online addictions concerns attachment style. This implies dispositional differences in the functioning of the attachment system and reflects cognitions and emotions, thus influencing different ways of interaction with acquaintances and strangers (Mikulincer and Shaver 2007). Internet addiction has been found to be associated with insecure attachment (Lin et al. 2011; Severino and Craparo 2013), with anxious and avoidant styles (Shin et al. 2009) and with dismissive and preoccupation attachment styles (Odacı and Çıkrıkçı 2014). Little attention has been paid to the association between attachment styles and other forms of online addiction (e.g., internet gaming disorder and social networking addiction). Recent studies have shown the predictive role of attachment in the excessive use of *Facebook* and online social network sites (Rom and Alfasi 2014; Yaakobi and Goldenberg 2014). More specifically, (i) securely attached individuals have larger social networks and more social ties (Jenkins-Guarnieri et al. 2012), (ii) anxiously attached individuals use *Facebook* more frequently and are constantly concerned about how they are perceived on *Facebook* (Lin 2015, 2016), and (iii) avoidant attached individuals show less interest in *Facebook* (Oldmeadow et al. 2013).

On the basis of the aforementioned findings and given that no empirical investigation has simultaneously considered the interrelationship of online addictions with dispositional factors, the present study investigated the extent to which identity styles and attachment orientations accounted for the three types of online addiction (i.e., internet addiction, online gaming addiction, and social networking addiction). The hypotheses of the current study were formulated taking into account the aforementioned socio-cognitive approach of identity formation (Berzonsky 1990) and the model of attachment style proposed by Feeney et al. (1994).

Feeney et al. (1994) developed a method to assess where each individual falls on two dimensions, *views of self* and *views of others*, and ranging from positive to negative or high to low. Four categories of attachment were delineated: (i) *secure* individuals have positive views of both self and others due to the responsive caregiving received in childhood; (ii) *preoccupied* individuals have a positive view of others and a negative view of self and strive for self-acceptance and approval of others; (iii) *fearful-avoidant* individuals have negative views of self and others, feel unloved and unlovable, and thus, they often avoid others to escape a possible rejection; and (iv) *dismissive-avoidant* individuals have positive views of self and negative views of others and reject and avoid other people to maintain their high sense of self (Bartholomew and Horowitz 1991; Feeney et al. 1994).

In light of these theoretical assumptions, it was expected that the online addictions would be associated (i) positively with preoccupation, fearful- and dismissive-avoidant attachment orientations and with diffuse-avoidant identity style and (ii) negatively with secure attachment orientation and with informational and normative identity styles. Moreover, in accordance with the transactional models of development (Bosma and Kunnen 2001; Grotevant 1987) and given the patterns of association between attachment and identity styles observed by Doumen et al. (2012), it was further expected (i) identity styles would be the primary variables for predicting online addictions, and (ii) attachment styles would contribute incrementally to this prediction.

## Method

### Participants and Procedure

Participants were recruited from Italian schools and universities and were voluntarily invited to participate in the study by completing a self-report offline questionnaire which took approximately 15 min to complete. A total of 712 questionnaires were collected. The mean age of participants was 21.63 years (SD = 3.90) with 381 males and 331 females. The sample was split into two age categories: those aged 16 to 19 years were classed as adolescents (*N* = 267; M_age_ = 18.22, SD = 1.04; M = 137, F = 130) and those aged over 20 years were classed as young adults (*N* = 445; M_age_ = 23. 67, SD = 3.55; M = 244; F = 201).

### Measures

#### Socio-Demographics

The questionnaire included demographic information concerning gender, age, and educational status.

#### Internet Addiction

The Italian version of the Internet Addiction Test (IAT; Young 1998; Fioravanti and Casale 2015) is a 20-item scale that assesses the severity of self-reported compulsive use of the Internet. Each item is rated on a 5-point Likert scale ranging from 1 (never) to 5 (always) leading to scores of between 20 and 100. Example items include “*How often do you find yourself anticipating when you will go online again*?” and “*How often do others in your life complain to you about the amount of time you spend online*?” The higher the score, the more likely someone has an Internet addiction with a score of 80 (out of 100) indicating that internet use is ‘severe’ and causing major problems in one’s life. In the present study, the internal reliability of the scale was excellent (Cronbach’s α = 0.96) and it was similar with values reported by Fioravanti and Casale (2015).

#### Internet Gaming Disorder

The Italian version of the nine-item Internet Gaming Disorder Scale-Short Form (IGDS9-SF; Pontes and Griffiths 2015; Monacis et al. 2016a) assesses the severity of IGD and its detrimental effects by examining both online and/or offline gaming activities occurring over a 12-month period. The scale comprises nine items corresponding to the nine core criteria defined by the DSM-5. They are answered on a 5-point Likert scale ranging from 1 (never) to 5 (very often) and examples of items are “*Have you lost interests in previous hobbies and other entertainment activities as a result of your engagement with the game*?” and “*Do you feel more irritability*, *anxiety*, *or even sadness when you try to either reduce or stop your gaming activity*?” Higher scores indicate a higher degree of gaming disorder. In the present study, the scale had an excellent reliability (Cronbach’s α = 0.96) and it was in line with the value reported by Pontes and Griffiths (2015).

#### Social Media Addiction

The Italian version of the Bergen Social Media Addiction Scale (BSMAS; Andreassen et al. 2016) assesses the experiences in the use of social media over the last year. It contains six items reflecting core addiction elements (Griffiths 2005). Each item is answered on a 5-point Likert scale ranging from 1 (very rarely) to 5 (very often). Examples of items are “*How long during the last year have you spent a lot of time thinking about social media or planned use of social media*?” and “*How long during the last year have you used social media so much that it has had a negative impact on your job/studies*?” In the present study, the internal consistency of the scale was very good (Cronbach’s α = 0.88) and was comparable with the finding reported in the original version (Andreassen et al. 2016).

#### Identity Styles

The Italian version of the Revised Identity Style Inventory (ISI-5; Berzonsky et al. 2013; Monacis et al. 2016c) assesses three identity styles, i.e., ‘informational,’ ‘normative,’ and ‘diffuse-avoidant.’ The scale comprises 36 items rated on a 5-point Likert scale (from 1 = Not at all like me to 5 = Very much like me). Sample items include: “*I handle problems in my life by actively reflecting on them*” for the 9-item informational scale; “*I think it is better to adopt a firm set of beliefs than to be open-minded*” for the 9-item normative scale; and “*Who I am changes from situation to situation*” for the 9-item diffuse-avoidant scale. The total score of each scale is computed by summing responses to the items. The internal consistency of the subscales was good (Cronbach’s α of 0.77 for informational style, 0.82 for the diffuse-avoidant style, and 0.62 for normative style). These values were comparable with those reported in the study carried out by Monacis et al. 2016b).

#### Attachment Style

The Italian version of the Attachment Style Questionnaire (ASQ; Feeney et al. 1994; Fossati et al. 2003) comprises 40 Likert-type items and assesses five styles of adult attachment related to two latent factors—anxiety and avoidance—formulated by Hazan and Shaver (1987) and Bartholomew (1990). There are five styles: confidence (C; 8 items) reflects the core aspects of secure attachment, i.e., attitudes of trust and positive expectations from self and others (e.g., “*I feel confident that other people will be there for me when I need them*”); discomfort with closeness (DwC; 10 items) reflects the role of withdrawal in avoidant attachment defined by Hazan and Shaver (1987) (e.g., “*I prefer to depend on myself rather than other people*”); need for approval (NfA; 7 items) reflects the need for acceptance and confirmation from others and characterizes Bartholomew’s (1990) conceptualization of anxious attachment (e.g., “*I wonder why people would want to be involved with me*”); preoccupation with relationship (PwR; 8 items) reflects a dependent approach to relationships according to Hazan and Shaver’s (1987) notion of anxious attachment (e.g., “*I often feel left out or alone*”); and relationship as secondary (RaS; 7 items) reflects Bartholomew’s (1990) concept of dismissive avoidant attachment (e.g., “*To ask for help is to admit that you are a failure*”). Each item is rated on a six-point scale, ranging from 1 (total disagree) to 6 (totally agree). Previous findings reported adequate internal consistency and test-retest reliability (Fossati et al. 2003). In the current study, Cronbach’s alpha values for the ASQ subscales ranged from 0.68 to 0.85.

### Ethics

The study procedures were carried out in accordance with the Declaration of Helsinki. The investigation was approved by the research team’s university ethics committee. Permission to conduct the research was required from heads of schools and institutions. Written informed consent was obtained from students over 18 years and from parents or legal guardians of students aged under 18 years.

### Statistical Analyses

Statistical analyses comprised an independent samples *t* test to verify gender and age effects on the scores of the dependent variables (internet addiction, social media addiction, and online gaming addiction). Bivariate correlation analyses were performed to analyze the pattern of associations between the variables of interest. The relationships between gender and the dependent variables were performed on the basis of point-biserial correlation coefficients. Causal relationships were examined by three hierarchical multiple regression analyses with the score of each addiction scale as dependent variable. Age and gender were included as independent variables in the first step, the three identity styles were introduced in the second step, and the five attachment styles were added in the third step.

## Results

### Descriptive Analyses

Mean scores and standard deviations for all variables are displayed in Table 1. With regard to gender, significant differences were found in the scores of the IGDS-SF9 and IAT for males (t[654,737] = 10.237, *p* < .001) and females (t[696,434] = 6.137, *p* < .001), whereas there were no differences in BSMAS scores. In addition, significant age differences between adolescents and adults emerged in the scores of all three online addictions: IGDS-SF9 (t[705,115] = −6.552, *p* < .001), BSMAS (t[665,629] = −5.561, *p* < .001), and IAT (t[660,963] = −4.084, *p* < .001).

**Table 1:** Mean and standard deviations of study variables (addiction scores, identity styles, and attachment styles)

	Total sample (*N* = 712)	Males (*N* = 381)	Females (*N* = 331)	Adolescents (*N* = 267)	Young adults (*N* = 445)
	Mean (SD)				
Technological addictions					
IGD9-SF	15.79 (8.87)	18.67 (9.77)	12.46 (6.24)	13.30 (6.38)	17.28 (9.78)
BSMAS	13.87 (5.67)	14.17 (6.26)	13.52 (4.90)	12.46 (4.68)	14.71 (6.05)
IAT	44.94 (18.27)	48.69 (19.96)	40.63 (15.03)	41.57 (15.35)	46.96 (19.56)
Identity styles					
Informational	34.21 (5.03)	33.81 (5.33)	34.66 (4.62)	33.13 (5.62)	34.85 (4.53)
Normative	26.59 (4.66)	26.50 (4.55)	26.70 (4.79)	26.19 (4.52)	26.84 (4.73)
Diffuse avoidant	22.68 (6.77)	23.24 (6.95)	22.03 (6.51)	22.65 (6.76)	22.69 (6.79)
Attachment styles					
Confidence	3.95 (.78)	3.92 (.83)	3.99 (.74)	4.00 (.73)	3.93 (.82)
Discomfort with closeness	3.84 (.83)	3.75 (.85)	3.94 (.79)	3.86 (.79)	3.83 (.85)
Need for approval	3.06 (.97)	3.12 (.94)	3.00 (.99)	3.06 (.96)	3.07 (.97)
Preoccupation with relationship	3.85 (.76)	3.75 (.72)	3.96 (.79)	3.88 (.77)	3.83 (.75)
Relationship as secondary	2.51 (1.01)	2.72 (1.00)	2.27 (.96)	2.49 (1.03)	2.52 (.99)

### Correlations Analyses

Table 2 shows the interrelationships among all variables. Findings demonstrated that IGD, SMA, and IA were positively associated. Scores on the three addiction scales also correlated positively with age, diffuse-avoidant style, DwC, RaS, NfA, and PwR and negatively with normative style and confidence. Among the online addictions, IA was negatively correlated with Informational style, and IGD and IA were negatively correlated with gender.

**Table 2:** Bivariate correlations between various variables and gaming, social networking, and internet use

	IGD9-SF	BSMAS	IAT
	Gaming	Social networking	Internet use
Sociodemographics			
Age	0.340**	0.259**	0.226**
Gender	−0.349**	0.057	−0.220**
Technological addictions			
BSMAS	0.752**	–	–
IAT	0.816**	0.817**	–
Identity styles			
Informational	−0.024	0.022	−0.077*
Normative	−0.169**	−0.092*	−0.156**
Diffuse avoidant	0.432**	0.441**	0.522**
Attachment styles			
Confidence	−0.494**	−0.442**	−0.512**
Discomfort with closeness	0.106**	0.196**	0.184**
Need for approval	0.427**	0.414**	0.491**
Preoccupation with relationship	0.075*	0.205**	0.193**
Relationship as secondary	0.434**	0.347**	0.470**

*p < .05; **p < .001

### Regression Analyses

#### Internet Addiction

Results indicated that Model 1 explained 8.6% of the variance in IA, *F*_2, 709_ = 33.54, *p* < .01. Model 2 explained an incremental 30% of the variance in the dependent variable score, *F*_5, 706_ = 87.35, *p* < .01, above and beyond the variance accounted for by socio-demographic characteristics. Model 3 explained an incremental 14% of the variance in the dependent variable score, *F*_10, 711_ = 75.58, *p* < .01, above and beyond the variance accounted for by identity styles and sociodemographic characteristics. The final model explained a total of 51.9% of the variance (*R* ^2^ adjusted = .512), and the *R* ^2^ change resulted in statistical significance at each step. The predictors were statistically significant except for the PwR attachment style. All *beta* coefficients are reported in Table 3.

**Table 3:** Regression coefficients between addiction scores (gaming, social networking, and internet use), age, gender, identity styles, and attachment styles

	IGD9-SF			BSMAS			IAT		
	B	S.E.	β	B	S.E.	β	B	S.E.	β
Gender^a^	−3.86	0.48	−0.22**	0.43	0.36	0.04	0.75	0.13	0.16**
Age	0.54	0.06	0.24**	0.31	0.05	0.22**	3.73	1.03	0.10**
Identity styles									
Informational	0.21	0.05	0.12*	0.12	0.04	0.11**	0.23	0.11	0.06*
Normative	−0.25	0.05	−0.13**	−0.09	0.04	−0.07**	−.48	0.11	−0.12**
Diffuse-avoidant	0.33	0.04	0.25**	0.24	0.03	0.28**	0.83	0.09	0.31**
Attachment styles									
Confidence	−3.23	0.37	−0.29**	−1.71	0.27	−0.24**	−5.94	0.78	−0.26**
Discomfort with closeness	−1.67	0.33	−0.16**	−0.57	0.25	−0.07**	−2.99	0.70	−0.14**
Relationship as secondary	1.18	0.30	0.13**	0.46	0.22	0.08*	2.73	0.63	0.15**
Need for approval	1.82	0.31	0.20**	0.97	0.23	0.17**	3.89	0.65	0.21**
Preoccupation with relationship	−0.67	0.35	−0.06	0.24	0.26	0.03	0.77	0.75	0.03

**p < .01; *p < .05

^a^1 = Male; 2 = Female

#### Internet Gaming Disorder

Results indicated that Model 1 including gender and age explained 20.6% of the variance in IGD, *F*_2, 709_ = 91.70, *p* < .01. Identity styles added in Model 2 explained an incremental 21.2% of the variance in the dependent variable score, *F*_5, 706_ = 101.41, *p* < .01, above and beyond the variance accounted for by sociodemographic characteristics. Attachment styles in Model 3 explained an incremental 13% of the variance in the dependent variable score, *F*_10, 711_ = 85.06, *p* < .01, above and beyond the variance accounted for by identity styles and sociodemographic characteristics. Therefore, the final model explained a total of 55% of the variance (*R* ^2^ adjusted = .542) and the *R* ^2^ change resulted in statistical significance at each step. The predictors were statistically significant, except for the attachment style of PwR. All *beta* coefficients are reported in Table 3.

#### Social Media Addiction

Results showed that Model 1 explained 6.8% of the variance in SMA, *F*_2, 709_ = 25.67, *p* < .01. Model 2 explained an incremental 22.2% of the variance in the dependent variable score, *F*_5, 706_ = 57.01, *p* < .01, above and beyond the variance accounted for by sociodemographic characteristics. Model 3 explained an incremental 9.2% of the variance in the dependent variable score, *F*_10, 711_ = 43.43, *p* < .01, above and beyond the variance accounted for by identity styles and sociodemographic characteristics. The final model explained a total of 38.3% of the variance (*R* ^2^ adjusted = .374), and the *R* ^2^ change resulted in statistical significance at each step. The predictors were statistically significant, except for gender and the PwR attachment style. All *beta* coefficients are reported in Table 3.

## Discussion

The aim of the present study was to simultaneously explore individual differences in online addictions by taking into account identity and attachment styles. Findings demonstrated that internet addiction, online gaming addiction (i.e., IGD), and social media addiction were interrelated and predicted by common underlying risk factors. Hierarchical multiple regressions showed that, among predictors, identity styles explained the greater amount of variance of the three online addictions, thus supporting their main role as protective or risk factors. The relationships between different addictive behaviors and their independent variables (age, gender, identity, and attachment styles) are discussed in the following subsections.

### Relationships between Different Online Addictions

Results from bivariate correlations showed high and positive associations between the three online addictions. More specifically, and as expected, internet addiction was similarly associated with IGD and social media addiction. These findings corroborate previous research (e.g., Andreassen et al. 2013, 2016; Monacis et al. 2016a; Sinatra et al. 2016) and provides empirical support for the concept of internet addiction as an *umbrella construct*, which comprises a wide range of online activities (Kuss and Billieux 2016), such as communication via social networking sites and playing online video games. Additionally, the high and positive association between IGD and social media addiction was probably due to the fact that many adolescents and young adults play games via social networking sites (Griffiths 2014) to receive pleasure, sense of accomplishment, etc. This also provides a route by which social networking games could represent a gateway to other potentially problematic leisure activities (Griffiths 2015).

### Demographic Factors

In general, both gender and age influenced online addictive behaviors. With regard to gender, being male was associated with both IGD and internet addiction. In line with other findings (e.g., Kuss et al. 2014; Andreassen et al. 2016; Monacis et al. 2016a), these results confirm males’ preference for these online activities, such as playing videogames, which can involve playing alone competitively. The lack of association between gender and social media addiction in the present study, is consistent with Sinatra et al.’s (2016) investigation but in contrast with other studies reporting contradictory findings such as higher SNS addiction scores in females (Andreassen et al. 2016; Monacis et al. 2016a; Pfeil et al. 2009) or higher SNS addiction scores in males (Raacke and Bonds-Raacke 2008). Another risk factor is represented by age, since young adults seem to be more engaged in online activities. However, unlike previous findings (Andreassen et al. 2016), this positive relationship may reflect the tendency of people to engage with more online technologies and applications as they age to satisfy their personal needs.

### Identity Style Formation

Findings showed that the construct of identity plays an important role in predicting online addictive behaviors. However, the predictive relationships were only partially confirmed, given the unexpected positive relationship between informational style and online addictions (i.e., as the informational style scores increased, online addiction levels significantly increased). Moreover, the relationship between informational style and social media addiction appears to be stronger than the relationships between this identity style and IGD and internet addiction. The influence in overusing social media may reflect the individual’s experience of a constant urge to check social networks, often considered as the best environment to find new information and updates and as useful tools to express and actualize an individual’s own identity either via self-display of personal information or via connections. On one hand, the informational style could be considered a significant risk factor for online addictions, along with other personality-related factors, such as extraversion which has been found to be a significant and positive predictor of internet addiction (Zamani et al. 2011) and of social networking addiction (Wang et al. 2014). On the other hand, this result is in contrast with studies showing the protective role of the informational style in the development of online addictive behaviors (e.g., Arabzadeh et al. 2012; Ceyhan 2010; Tabaraei et al. 2014).

As expected, normative style was a protective factor for all three online addictions. Indeed, normative-oriented individuals, in internalizing significant others’ expectations and values, tend to protect and conserve their own identity structure by reducing the use of technological communication. In this case, the virtual environment could represent an uncertain space characterized by a variety of identities and values. This negative relationship is in line with the findings reported by other researchers (e.g., Arabzadeh et al. 2012; Ceyhan 2010; Tabaraei et al. 2014; Sinatra et al. 2016). Finally, the diffuse-avoidant style is a risk factor in determining online addictions. Individuals with a diffuse-avoidant style tend to avoid or procrastinate identity conflicts, probably through an excessive use of internet, social networks sites, and videogames. In other words, individuals who need to escape from real-life situations prefer to display their self-expression in *open* and virtual environments, characterized by the maintenance of established offline networks and by the manifold online ties that are indicative of bridging rather than bonding social capital. These findings give further support to previous reported findings (White et al. 2003; White et al. 1998; Hojjat et al. 2015).

Another noteworthy observation is that, from the examination of the standardized regression coefficients of the two identity risk factors (informational and diffuse-avoidant style), the diffuse-avoidant style appears to have a more predictive power in all three online addictions than the informational style. This aspect stresses the potential role played by diffuse-avoidant style as the most negative identity risk factor in the development of online addictions.

### Attachment Styles

As expected, attachment styles are important dispositional factors in determining online addictive behaviors. Indeed, similarly to previous research, the findings of the present study generally supported the aforementioned hypotheses regarding the causal relationship between these constructs. More specifically, secure attachment orientation negatively predicted the three online addictive behaviors. Individuals characterized by high self-esteem and enjoyment in intimate relationships and in sharing feelings with others (Bowlby 1969/1982, 1973, 1980) may be lower at risk of becoming addicted to online gaming, social networking, and the internet. The negative regression coefficients of the confidence style confirm its expected function, since it is a protective factor against addictions. However, the negative relationship between secure attachment and social networking addiction is inconsistent with other findings where securely attached individuals, being able to manage SNSs, use them as a tool to have more social ties with others, thus increasing their sense of social belonging and interpersonal competency (Jenkins-Guarnieri et al. 2012; Oldmeadow et al. 2013; Pempek et al. 2009).

The expected positive associations were found between need for approval (referred to as an anxious style) and all three online addictions. Individuals with this attachment tendency, showing excessive desires and efforts for acceptance and being dependent from others, are more likely to use technologies to obtain approval and positive feedback, therefore putting themselves at risk of addiction. Moreover, individuals with relationships as secondary style, referred to an avoidant attachment, are led by their attachment attitudes to assess dismissive approaches to close relationships and tend to satisfy their need of social belonging by using the online format, which affords such a dismissive approach to close relationships by maintaining a ‘safe’ distance from others, thus developing at-risk addictive behaviors.

On the other hand, the discomfort for intimacy style (referred to an avoidant style) represents a protective factor, since individuals unable to invest in intimacy and to share feelings, thoughts, and emotions with others tend to reduce any online activity. Consistent with other findings, this style is associated with less interest in the use of *Facebook* and other social networks (Oldmeadow et al. 2013). Finally, no significant associations emerged between preoccupation with relationships and addiction scores, thus contrasting findings reported by Schimmenti et al. (2014). This contradictory result may depend on the psychological characteristics of the previous Italian sample of participants, who, suffering from childhood experiences of emotional, physical, and sexual abuse, tended to use the Internet as a virtual retreat in order to protect themselves from feelings of loneliness and fears about real interactions.

The present study is not without its limitations. First, much caution should be taken in the interpretation of these findings, given that participants were sampled on the basis of a self-selected convenience sampling strategy. Further studies should consider a more representative sample of the population in order to generalize the findings. Second, the use of a self-report questionnaire is associated with well-known biases (e.g., social desirability and recall biases). Future investigations should not only include a multi-method assessment of identity styles, attachment styles, and online addictions but also use longitudinal designs in order to better assess the directionality between the causal relationships. Taken as a whole, as far as the authors are aware, this is the first study to investigate individual differences in the interrelationships between three online addictions, by integrating the theory of identity formation and attachment style. Despite the exploratory nature of the present study, it adds an innovative contribution to the existing literature.
